# Supplementary material for: Diversify the syllabi: Underrepresentation of female authors in college course readings
Source: PLoS One. 2020 Oct 28;15(10):e0239012. doi: 10.1371/journal.pone.0239012 (PMC7592743; doi:10.1371/journal.pone.0239012)
Supplement: S1 Codebook — (DOCX) [file pone.0239012.s005.docx]

Diversify the syllabi: Underrepresentation of female authors in college course readings

Documentation for 20200713_deidentified_reading_level_data.csv

**Data report overview:**

| Feature | Result |
| --- | --- |
| Number of observations | 2671 |
| Number of variables | 88 |

**Variable Groups**

**Course Characteristics**

| Variable | Class | # unique values | Description |
| --- | --- | --- | --- |
| course_id | character | 148 | Unique course identifier |
| school_name | factor | 6 | School name |
| dept_area_code | character | 40 | Department area code |
| category | factor | 4 | Discipline category |
| se_cur_enr | number | 54 | Course enrollment |
| syllabi_central | factor | 2 | Did syllabus come from archival data source |
| division | factor | 2 | Course level |

**Reading Characteristics**

| Variable | Class | # unique values | Description |
| --- | --- | --- | --- |
| reading | factor | 2671 | Reference for reading assignment |
| reading_type_clean | factor | 4 | Type of reading |

**Instructor(s) Gender**

| Variable | Class | # unique values | Description |
| --- | --- | --- | --- |
| gender.instructor | factor | 5 | Gender of instructor |
| gender.instructor2 | factor | 5 | Gender of second instructor |
| gender.instructor3 | factor | 5 | Gender of third instructor |
| gender.instructor4 | factor | 5 | Gender of fourth instructor |
| gender.instructor5 | factor | 5 | Gender of fifth instructor |
| gender.instructor6 | factor | 5 | Gender of sixth instructor |
| gender.instructor7 | factor | 5 | Gender of seventh instructor |

**Author(s) Gender**

| Variable | Class | # unique values | Description |
| --- | --- | --- | --- |
| gender.first.author | factor | 5 | Gender of first author |
| gender.last.author | factor | 5 | Gender of last author |
| gender.other.author | factor | 5 | Gender of first middle author |
| gender.other.2 | factor | 5 | Gender of second middle author |
| gender.other.3 | factor | 5 | Gender of third middle author |
| gender.other.4 | factor | 5 | Gender of fourth middle author |
| gender.other.5 | factor | 5 | Gender of fifth middle author |
| gender.other.6 | factor | 5 | Gender of sixth middle author |
| gender.other.7 | factor | 5 | Gender of seventh middle author |
| gender.other.8 | factor | 5 | Gender of eighth middle author |
| gender.other.9 | factor | 5 | Gender of ninth middle author |
| gender.other.10 | factor | 5 | Gender of tenth middle author |
| gender.other.11 | factor | 5 | Gender of eleventh middle author |
| gender.other.12 | factor | 5 | Gender of twelfth middle author |
| gender.other.13 | factor | 5 | Gender of thirteenth middle author |
| gender.other.14 | factor | 5 | Gender of fourteenth middle author |
| gender.other.15 | factor | 5 | Gender of fifteenth middle author |
| gender.other.16 | factor | 5 | Gender of sixteenth middle author |
| gender.other.17 | factor | 5 | Gender of seventeenth middle author |
| gender.other.18 | factor | 5 | Gender of eighteenth middle author |
| gender.other.19 | factor | 5 | Gender of nineteenth middle author |
| gender.other.20 | factor | 5 | Gender of twentieth middle author |
| gender.other.21 | factor | 5 | Gender of twenty-first middle author |
| gender.other.22 | factor | 5 | Gender of twenty-second middle author |
| gender.other.23 | factor | 5 | Gender of twenty-third middle author |
| gender.other.24 | factor | 5 | Gender of twenty-fourth middle author |
| gender.other.25 | factor | 5 | Gender of twenty-fifth middle author |
| gender.other.26 | factor | 5 | Gender of twenty-sixth middle author |
| gender.other.27 | factor | 5 | Gender of twenty-seventh middle author |
| gender.other.28 | factor | 5 | Gender of twenty-eighth middle author |
| gender.other.29 | factor | 5 | Gender of twenty-ninth middle author |
| gender.other.30 | factor | 5 | Gender of thirtieth middle author |
| gender.other.31 | factor | 5 | Gender of thirty-first middle author |
| gender.other.32 | factor | 5 | Gender of thirty-second middle author |
| gender.other.33 | factor | 5 | Gender of thirty-third middle author |
| gender.other.34 | factor | 5 | Gender of thirty-fourth middle author |
| gender.other.35 | factor | 5 | Gender of thirty-fifth middle author |
| gender.other.36 | factor | 5 | Gender of thirty-sixth middle author |
| gender.other.37 | factor | 5 | Gender of thirty-seventh middle author |
| gender.other.38 | factor | 5 | Gender of thirty-eighth middle author |
| gender.other.39 | factor | 5 | Gender of thirty-ninth middle author |
| gender.other.40 | factor | 5 | Gender of fortieth middle author |
| gender.other.41 | factor | 5 | Gender of forty-first middle author |
| gender.other.42 | factor | 5 | Gender of forty-second middle author |
| gender.other.43 | factor | 5 | Gender of forty-third middle author |
| gender.other.44 | factor | 5 | Gender of forty-fourth middle author |
| gender.other.45 | factor | 5 | Gender of forty-fifth middle author |
| gender.other.46 | factor | 5 | Gender of forty-sixth middle author |
| gender.other.47 | factor | 5 | Gender of forty-seventh middle author |
| gender.other.48 | factor | 5 | Gender of forty-eighth middle author |
| gender.other.49 | factor | 5 | Gender of forty-ninth middle author |
| gender.other.50 | factor | 5 | Gender of fiftieth middle author |
| gender.other.51 | factor | 5 | Gender of fifty-first middle author |
| gender.other.52 | factor | 5 | Gender of fifty-second middle author |
| gender.other.53 | factor | 5 | Gender of fifty-third middle author |
| gender.other.54 | factor | 5 | Gender of fifty-fourth middle author |
| gender.other.55 | factor | 5 | Gender of fifty-fifth middle author |
| gender.other.56 | factor | 5 | Gender of fifty-sixth middle author |
| gender.other.57 | factor | 5 | Gender of fifty-seventh middle author |
| gender.other.58 | factor | 5 | Gender of fifty-eighth middle author |
| gender.other.59 | factor | 5 | Gender of fifty-ninth middle author |
| gender.other.60 | factor | 5 | Gender of sixtieth middle author |
| gender.other.61 | factor | 5 | Gender of sixty-first middle author |
| gender.other.62 | factor | 5 | Gender of sixty-second middle author |
| gender.other.63 | factor | 5 | Gender of sixty-third middle author |
| gender.other.67 | factor | 5 | Gender of sixty-seventh middle author |
| gender.first.last.author | character | 12 | Gender of first and last authors |
| femPercAuth | numeric | 29 | Percent female authors for reading |
| femSumAuth | number |  | Number of female authors for reading |
| hasUnkAuth | character | 2 | Does reading have unknown author(s)? |
| hasNonBiAuth | character | 2 | Does reading have non-binary author(s)? |

Documentation for 20200427_NSF_2017_college_graduates.csv

| Variable | Class | # unique values | Description |
| --- | --- | --- | --- |
| sex | factor | 2 | Unique course identifier |
| major_field | factor | 4 | Broad category of field |
| minor_field | factor | 36 | Detailed category of field |
| discipline | factor | 4 | Discipline category of field |

Documentation for 20200427_earned_doctorates_2018_discipline.csv

| Variable | Class | # unique values | Description |
| --- | --- | --- | --- |
| field | factor | 4 | Discipline category of field |
| male | number |  | Number of males earned doctorates |
| female | number |  | Number of females earned doctorates |
