# Supplementary material for: Diversify the syllabi: Underrepresentation of female authors in college course readings
Source: PLoS One. 2020 Oct 28;15(10):e0239012. doi: 10.1371/journal.pone.0239012 (PMC7592743; doi:10.1371/journal.pone.0239012)

S1 Appendix. Reading types by discipline and author gender.

**Table. Count of reading types by discipline.**

## # A tibble: 15 x 3
## # Groups: category [4]
## category reading_type_clean count
## <chr> <chr> <int>
## 1 Humanities book 467
## 2 Humanities book chapter 6
## 3 Humanities journal article 195
## 4 Humanities other 440
## 5 Other book 83
## 6 Other book chapter 4
## 7 Other journal article 99
## 8 Other other 62
## 9 Social Sciences book 261
## 10 Social Sciences book chapter 38
## 11 Social Sciences journal article 500
## 12 Social Sciences other 126
## 13 STEM book 77
## 14 STEM journal article 20
## 15 STEM other 11

**Figure. Percentage of readings by type and author gender across disciplines.**


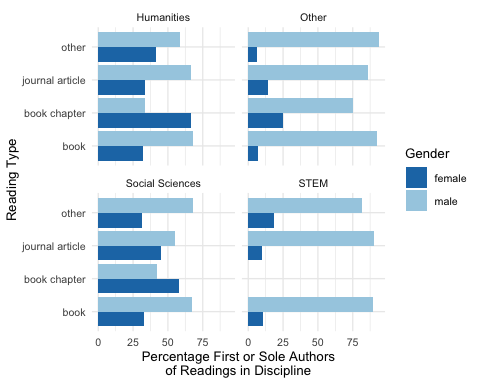

Supplement: S1 Appendix — (DOCX) [file pone.0239012.s007.docx]
