# Supplementary material for: Diversify the syllabi: Underrepresentation of female authors in college course readings
Source: PLoS One. 2020 Oct 28;15(10):e0239012. doi: 10.1371/journal.pone.0239012 (PMC7592743; doi:10.1371/journal.pone.0239012)

S2 Appendix. Comparison of syllabi from archival sources with syllabi received from faculty via email.

**The text of the email sent to faculty requesting syllabi introduced the topic of the project this way:**

“I am working with a small team to conduct a project (IRB ID 202001078) that answers the question: To what extent are faculty at Washington University in St. Louis including readings by women in their syllabi? There is little existing work on the topic of women authors on university syllabi. However, a few studies in a few fields have found that readings with female authors are included less frequently in course syllabi than readings with male authors, at least in some fields.”

**Table. Count and percentage of syllabi with female and male first authors by discipline and syllabus source.**

## # A tibble: 16 x 5
## # Groups: category, syllabi_source [8]
## category syllabi_source gender.first.author count percentage
## <chr> <chr> <chr> <int> <dbl>
## 1 Humanities no female 306 44.0
## 2 Humanities no male 390 56.0
## 3 Humanities yes female 98 22.6
## 4 Humanities yes male 336 77.4
## 5 Other no female 25 11.0
## 6 Other no male 203 89.0
## 7 Other yes female 2 7.69
## 8 Other yes male 24 92.3
## 9 Social Sciences no female 118 46.1
## 10 Social Sciences no male 138 53.9
## 11 Social Sciences yes female 258 37.8
## 12 Social Sciences yes male 425 62.2
## 13 STEM no female 4 18.2
## 14 STEM no male 18 81.8
## 15 STEM yes female 11 12.2
## 16 STEM yes male 79 87.8

**Figure. Percentage of readings with female first or sole author for syllabi from archival and emailed sources by discipline.**


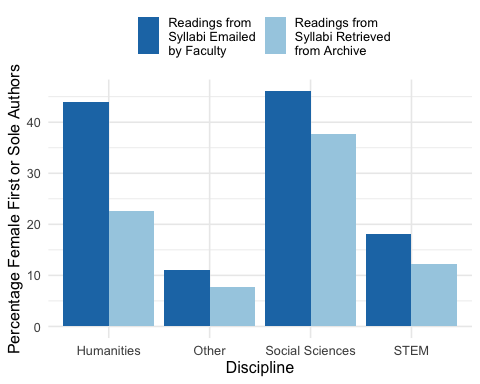

Supplement: S2 Appendix — (DOCX) [file pone.0239012.s008.docx]
