# Supplementary material for: Diversify the syllabi: Underrepresentation of female authors in college course readings
Source: PLoS One. 2020 Oct 28;15(10):e0239012. doi: 10.1371/journal.pone.0239012 (PMC7592743; doi:10.1371/journal.pone.0239012)
Supplement: Attachment — Submitted filename: PLOS_June.2020.pdf [file pone.0239012.s009.pdf]

PONE-D-20-13578
Diversify the syllabi: Underrepresentation of female authors in college course readings
PLOS ONE

Recommended is theoretical and empirical development for this manuscript.

1) Theoretically, the framework appears to that of "gender stratification." Addressing the analytical issues within the framework will set a context for the paper and develop an argument.

2) Empirically, the manuscript will be developed by considering the women authors in readings, by levels of courses and types of readings. These, in turn, will link to broader issues (above) of gender stratification.

a) More specifically, address women authors
1) appearing in syllabi in these levels of courses:
-Undergraduate courses: lower-division
-Undergraduate courses: upper-division
-Graduate courses

2) and appearing in text books compared to readings outside of textbooks.

Retain analyses, by field, for 1) and 2)

b) In implications:
1) Assigned readings in upper division, and especially graduate-level courses, have more bearing on modeling for scholarship and workforce diversity (linked to gender stratification).

2) Assigned reading that appear in scholarly journals and books (compared to textbooks) have more bearing on modeling for scholarship and workforce diversity (linked to gender stratification).

3) In addition, for continuing research (not necessarily that for present manuscript):
Particularly revealing would be analysis of readings, by gender of author, that appear in reading lists for doctoral exams. These are influential for career-bound students.
